# Open conversion for laparoscopically difficult cholecystectomy is still a valid solution with unsolved aspects

**DOI:** 10.1186/s13017-019-0227-4

**Published:** 2019-02-18

**Authors:** M. Mannino, A. Toro, M. Teodoro, F. Coccolini, M. Sartelli, L. Ansaloni, F. Catena, I. Di Carlo

**Affiliations:** 1 Department of Surgical Sciences and Advanced Technologies “GF Ingrassia”, Cannizzaro Hospital, University of Catania, Via Messina, 829, 95126 Catania, Italy; 2 Department of Surgery, Augusta Hospital, Augusta, SR Italy; 3 General, Emergency and Trauma Surgery Department, Bufalini Hospital, Cesena, Italy; 4 Department of Surgery, Macerata Hospital, Macerata, Italy; 5 Emergency Surgery, Parma Hospital, Parma, Italy

**Keywords:** Open conversion, Difficult cholecystectomy, Laparoscopy

## Abstract

The difficult laparoscopic cholecystectomy remains a surgical challenge for surgeons who must decide between laparoscopic continuation and open conversion. The balance between the lack of open surgery training of young surgeons and the risk of maintaining the laparoscopic approach in difficult laparoscopic cholecystectomy is still an unresolved problem. Furthermore, the time that must be spent in an attempt to complete laparoscopic surgery before conversion is still controversial. The authors in this letter discuss about these and other questions that still require an answer.

## Background

The multiple guidelines [1–4] that have tried in the past to solve the problem of difficult laparoscopic cholecystectomies have not solved the problem right now. Multiple aspects are unsolved such as when a procedure has to be converted, after how much, and when a patient has to be operated in open surgery. As no answers until now, the authors try with this letter to propose some questions that concern the problem in order to stimulate further studies that can help both surgeons and patients.

Laparoscopic surgery has completely changed the clinical course of some surgical procedures. Among these procedures, laparoscopic cholecystectomy has had a revolutionary impact, changing the clinical management of related diseases forever.

Although laparoscopy as a mandatory procedure has almost erased open surgery, difficult cholecystectomy due to acute cholecystitis still represents an actual challenge for surgeons performing laparoscopy. In these cases, the surgeon has to cure the patient and has to avoid complications that can worsen the patient’s quality of life forever. For this reason, the kind of procedure that should be applied in cases of difficult acute cholecystitis is still undetermined.

In fact, all the cases in which the Calot triangle cannot be approached safely due to the absence of a critical view of safety (CVS) require a different approach [1]. However, whether this approach has to be done by laparoscopy, by open surgery or by conversion during laparoscopy, as well as when to perform the approach, is still up for discussion. A couple of consensuses have been agreed upon (at minimum twice), establishing some of the points but not solving all of the issues [1, 2].

The most cited consensus is that of Tokyo (TG18), which has established guidelines. This manuscript states that the surgeons that persevere laparoscopically, in cases of difficult acute cholecystitis, have increased in number; at the same time, this manuscript stated that conversion needs to be applied when the CVS is not achievable. The majority of surgeons participating in the Tokyo guidelines are in the laparoscopic field. The question that remains is how many of the surgeons are prone to go on in laparoscopic surgery and until when. In addition, how many of them convert immediately when the condition does not allow them to continue the laparoscopy?

The TG18 states that only biliary leakage is increased with the laparoscopic technique, but all other remnant complications are the same in relation to open surgery. This statement has a double citation; indeed, if we look at the first citation, the article analyzes a mixed population for a very long period, in which the patients were operated on only by open surgery [5]. The second citation analyzes the problem of young surgeons who, due to the almost comprehensive approach of surgery by laparoscopy, are not sufficiently trained in laparoscopy. Consequently, for this younger generation, the visibility in open surgery led to an increased risk of biliary duct injuries (BDI) [6]. Thus, the supposed major number of complications by open surgery is not demonstrated and needs to be re-discussed. All citations, however, agree on the fact that the adverse event “bile leakage” is more common in laparoscopic surgery performed in emergency, which is, in our opinion a dangerous adverse effect because it can mask an underlying BDI.

Surgeons around the world, after an initial period in which the majority of them were convinced that the procedure had to be completed by laparoscopy, realized that the procedure had to be converted to avoid all complications. This realization was because difficult cholecystectomy had a stable number of complications in open surgery compared to laparoscopy. [7]

An interesting study shows that, in the last 15 years, after an initial attempt to complete every procedure by laparoscopy, the conversion rate increased and has remained stable for the last 10 years. [7]

Many manuscripts have proposed scores that indicate the possibility a patient needs to be converted [8] or that propose if they have to be operated on directly by open surgery. However, currently, no algorithm exists, especially when open conversion has to be applied [9].

A decisional algorithm has been reported focusing on the Mirizzi syndrome. The authors, however, do not focus on all acute difficult cholecystitis. Furthermore, the general management is investigated, leaving lack in the operative setting [10].

In all the papers published, the absence of CVS is the most important factor considered for conversion. The factors that are able to determine this absence of CVS include a complete buried gallbladder, an impacted stone and the incapability to retract the gallbladder. All these factors are mandatory indicators for open conversion in cases of difficult cholecystectomy for acute cholecystitis, because even a subtotal laparoscopic cholecystectomy does not appear technically feasible in the presence of these conditions [1, 9]. In our opinion, the impossibility of grasping the gallbladder is the condition that most of all make technically not possible to perform a subtotal laparoscopic cholecystectomy, while a complete buried gallbladder and the absence of CVS make the laparoscopic approach dangerous, determining a higher risk to cause a biliary lesion. It still remains an issue to determine the following prior to performing surgery: which patients have to be operated on directly by open surgery and which patients have to be subjected to laparoscopy tentatively. [11, 12]

Strictly related to open conversion, the question that arises is how long will the surgeon have to try to complete the laparoscopy before conversion? Currently, there is no time limit because no manuscripts have studied this aspect.

A manuscript exists in which surgeons from Korea, Japan and Taiwan were interviewed; it was determined that beginning 60 min prior to surgery, there are some surgeons who do not consider any limits for this surgery [13]. This aspect has to be studied because prolongation of the procedure presents an increased risk for morbidity, especially in benign disease, that in an expert’s hands does not surpass 30 min. The majority of these procedures are done by residents, and this fact represents another aspect to be studied. Difficult cases have to be managed or helped by expert surgeons (not only hepatobiliary surgeons, but by any surgeon with a high laparoscopic background) to establish the limit for the tentative completion of laparoscopy; however, to avoid exceeding a previously established time limit, the conversion needs to be adopted without hesitation. Therefore, this manuscript describes that the number of risk factors can predict the risk of the operation being prolonged for more than 90 min. Therefore, the decision to convert has to be made quickly to avoid the risk of prolongation of the procedure and the consequent risk of morbidity [14]. It is important to remind that the patients treated with a converted open approach are often the ones with the poorest performance status previous to surgery or the ones in the most serious conditions due to a septic status given by the cholecystitis. In any case, these patients benefit from a quick surgical approach.

So, in order to establish a time limit parameter, it should be considered an ideal scenario in the hand of solely expert surgeons, not considering the time spent on the same kind of surgery by least experienced operators.

In this particular pathologic situation, a time limit is lacking and, looking at the answers given by surgeons, open to very different evaluations. We think every surgeon should have a model to refer on, but with the possibility, due to the anatomic situation, the performance status of the patient or any other variable, to diverge from the ideal result.

An intra-operative cholangiography can be useful in some cases to avoid conversion, but we have to consider if the requested prolongation of times can be tolerated by the patient we are operating and that in some cases, it is not technically feasible due to the fact that the cystic duct is not always clearly recognizable in case of a diffuse inflammation [15].

Of course, cholecystostomy has to be considered in case of frail patients and substantially is out of this discussion because these patients cannot be submitted initially to surgery.

When open conversion is adopted, many procedures are described. The most commonly used procedure is subtotal cholecystectomy [16, 17]. This technique considers the suture of the infundibulum, 1 cm from the cystic duct [17]. A different technique, called fenestrating, has also been applied. In this technique, the gallbladder can remain in situ, especially in the posterior wall, and the cystic duct can be ligated from inside the gallbladder. However, this last maneuver represents a considerable risk due to the proximity of the common bile duct and to the consequent possibility of stenosis by the retraction of this duct [18]. When subtotal cholecystectomy is applied, particular attention has to be reserved for the length of the infundibulum, which is sutured in place. If 3–4 cm of infundibulum is left in place, the risk of leaving small stones or the risk of new stone formation in this new generated pouch represents an issue requiring a re-operation [18]. In the case of a subtotal cholecystectomy, the distance of 1 cm from the infundibulum has to be respected to prevent all these complications [17, 19].

Finally, converted patients are the poorest fairing that surgeons are unable to complete laparoscopy on. It could be advisable to screen these patients pre-operatively (Is it a difficult gallbladder? Is an open approach better?). As an algorithm does not exist currently, in cases of difficult laparoscopic approaches, the surgeon has to recognize a difficult gallbladder in the intra-operative setting as soon as possible, trying to avoid maneuvers that can damage the CBD. The best method could be to set a time limit and to be ready to change the approach. Of course, the surgeons have to use the technique that they know best, but subtotal cholecystectomy is the technique that best preserves patients from complications. Some surgeons experienced the conversion as a failure in their practice; however, thinking in terms of long-term results, this approach can be a success for both the surgeon and the patient.

## Conclusions

Although laparoscopic cholecystectomy is nowadays one of the most performed surgical operation in abdominal surgery, some aspects, concerning the emergency setting, have to be yet investigated.

A valid algorithm which can be used in the presence of acute cholecystitis to decide pre- or intra-operatively the best approach is still lacking. Furthermore, when open conversion is needed, the time before to convert still do not have a full consensus and need to be further explored.

## Abbreviations

BDI: Biliary duct injuries
CVS: Critical view of safety
TG18: Tokyo guidelines 2018
